# Skin Cancer Knowledge, Beliefs, Self-Efficacy, and Preventative Behaviors among North Mississippi Landscapers

**DOI:** 10.1155/2013/496913

**Published:** 2013-10-07

**Authors:** Vinayak K. Nahar, M. Allison Ford, Jeffrey S. Hallam, Martha A. Bass, Amanda Hutcheson, Michael A. Vice

**Affiliations:** ^1^Department of Health, Exercise Science & Recreation Management, University of Mississippi, 215 Turner Center, P.O. Box 1848, Oxford, MS 38677, USA; ^2^Department of Social and Behavioral Sciences, College of Public Health, Kent State University, 750 Hilltop Drive, Kent, OH 44242, USA

## Abstract

There are slightly over one million workers in the landscape service industry in the US. These workers have potential for high levels of solar ultraviolet radiation exposure, increasing their risk of skin cancer. A cross-sectional sample of 109 landscapers completed a self-administered questionnaire based on Health Belief Model (HBM). The participants correctly answered 67.1% of the knowledge questions, 69.7% believed they were more likely than the average person to get skin cancer, and 87.2% perceived skin cancer as a severe disease. Participants believed that the use of wide-brimmed hats, long sleeved shirts/long pants, and sunscreen was beneficial but reported low usage of these and other sun protective strategies. The primary barriers to using sun protection were “I forget to wear it” and “it is too hot to wear.” Of the HBM variables, perceived benefits outweighing perceived barrier (*r* = .285, *P* = .003) and self-efficacy (*r* = .538, *P* = .001) were correlated with sun protection behaviors. The reasons for absence of the relationship between perceived skin cancer threat and sun protection behaviors could be lack of skin cancer knowledge and low rate of personal skin cancer history.

## 1. Introduction

In the US, there are millions of workers in the outdoor occupations who have the potential for overexposure to solar ultraviolet radiation (UVR), placing them at higher risk of developing skin cancer [1, 2]. Outdoor workers are generally exposed to UVR during work for two to eight hours which far exceeds recommended guidelines [3, 4]. Additionally, a vast majority of outdoor jobs in the US are held by fair skinned individuals who are increasingly at risk of skin cancer [5]. However, several studies report that some outdoor workers are engaging in sun protection practice but a large population of outdoor workers continues to show inadequate levels of sun protection [6]. This use of inadequate levels of sun protective behaviors amongst outdoor workers could be due to many of the outdoor workers being males, who often engage in lower levels of sun protection behaviors than females [5].

A review article showed that a number of research studies were conducted on outdoor workers to assess quantitative data of sun exposure and sun protection behaviors, with the majority of studies carried out on farmers and recreation workers [6]. Nonetheless, far too little attention was given to members of other occupational groups that work outdoors, such as landscapers. It was estimated in the US that there are slightly over one million workers in the landscape service industry [7]. To best of our knowledge, to date, no US based study has targeted landscapers, one of the most common outdoor workers [1]. Thus, it is extremely important to study landscapers' behaviors regarding sun exposure and sun protection, and determine their skin cancer risk perception and how these perceptions and other cognitive factors are associated with their sun protection practices. Such information could be best achieved with theoretically driven research, since theories help explain the structural and psychological determinants of behavior and provide direction for how to develop more effective ways to achieve behavior change [8].

For this study, the Health Belief Model (HBM) was used as a theoretical framework to explain and assess why landscapers may or may not take action to practice sun protection behaviors. The HBM is one of the most extensively used theories that was developed in the early 1950s by Hochbaum, Kegels, and Rosenstock [8]. The constructs of HMB include perceived threat that consists of two parts: perceived susceptibility and perceived severity. The former refers to one's belief about probability of the risk of contracting a disease or condition. The construct of perceived severity is an individual's belief about the seriousness of the disease. Perceived threat determines if the individual is likely to take health-related action. The next construct of this model is perceived benefits which addresses the belief of an individual about values or usefulness of a new behavior to reduce threat of illness. Perceived barrier is one's evaluation of obstacles in his/her way that may prevent or hinder the engagement in new behavior. The behavior change is likely to occur if the perceived benefits outweigh the perceived barriers. Cues to action are stimuli which may be internal (e.g., symptoms) or external (e.g., events, people, and flyers) that increase motivation of people to change their behavior. Self-efficacy refers to strong belief about one's ability to successfully execute a particular behavior required to produce the desired outcome [9]. Modifying factors such as demographic, sociopsychological, and structural factors (i.e., knowledge about the disease and prior contact with the disease) may indirectly influence health-related behavior by affecting an individual's perceived threat.

The present study focused specifically on landscape service workers of North Mississippi. The primary purpose of this study was to determine and explain landscapers' health beliefs with regard to skin cancer, level of skin cancer knowledge, cues to sun protective actions, self-efficacy to engage in sun protection practices, and current sun protection behaviors. Moreover, this study examined the utility of HBM constructs in explaining landscapers' engagement in sun protection behaviors.

By assessing the level of skin cancer knowledge, perceptions of skin cancer, and frequency of sun protection measures use among landscapers of North Mississippi, public health professionals will gain valuable insights regarding development, implementation, and evaluation of interventions to prevent skin cancer in this population. In addition, the information related to barriers to sun protection will provide a deeper understanding of how to modify or design sun protection intervention strategies that will well match the specific needs of landscapers which will ultimately help in changing their sun protection behaviors and reduce the risk and rates of skin cancer among this high risk target groups.

## 2. Methods

### 2.1. Study Design and Procedure

This cross-sectional study was conducted between May and October 2012. After Institutional Review Board approval, the landscape service companies in North Mississippi were identified through internet search and personal contacts. A phone call was made to the landscaping service companies to obtain consent, and companies were given choices to participate either through on-site administration of the survey or self-addressed prepaid postage survey. The lead investigator hand-delivered the information letter and questionnaire in a confidential envelope to participants during breaks. The survey took approximately ten minutes to complete. Moreover, the information letter and questionnaires with a self-addressed prepaid postage envelope were sent to the companies that decided to participate with this approach. The participants voluntarily completed the questionnaires and sent them back to the lead investigator in the enclosed self-addressed prepaid postage envelope. 

### 2.2. Measurement Instrument

 The modified version of “Skin Cancer Survey” was used for this study [10]. The questionnaire items which were added to the “Skin Cancer Survey” were derived from previous studies [11–16]. The modified self-reported questionnaire included 41 items on sociodemographic information, sun protection behaviors, knowledge, and HBM constructs. The instrument was reviewed by a dermatologist for the validity. The reliability coefficient scores for each scale are as follows: knowledge *α* = .83, health beliefs (perceived risk, perceived benefits, and perceived barrier) *α* = .81, cues to action *α* = .82, and self-efficacy *α* = .55.

### 2.3. Measures

 To determine sun protection behaviors, participants were asked to indicate on a five-point Likert-type scale (never, rarely, sometimes, frequently, and always) how frequently they use sun protection measures when out in the sun for 15 minutes or more. Moreover, after every sun protection behavior question, participants were asked to select barriers if they do not always perform the sun protection behaviors. All responses to barriers were measured using Nominal scale (checked = yes; not checked = no). Additionally, respondents were given an option if they wish to specify any other barriers for not always practicing sun protection behavior. 

 The knowledge of participants regarding skin cancer was assessed via the use of 10 items and was evaluated based on correct response. All the items required Nominal level responses (true, false, and I do not know). Responses false and I do not know were considered incorrect. 

The participants' perceived susceptibility to skin cancer was measured by two items on five-point Likert-type scale anchored with strongly disagree, disagree, neutral, agree, and strongly agree. Two items assessed participants' perceived severity of skin cancer. The response metric was on five-point Likert-type scale ranging from strongly disagree to strongly agree. The scores from perceived susceptibility and perceived severity were multiplied to obtain the perceived threat score.

 Perceived benefits of sun protection were measured by using six Likert-type items with five-point responses ranging from strongly disagree to strongly agree. One item was used to measure perceived barrier to sun protection with responses on a 5-point Likert-type scale ranging from strongly disagree to strongly agree. A variable perceived benefits outweighing perceived barrier was created by average of perceived benefits score minus perceived barrier score.

 Cues to action were measured by asking participants about their sources of sun protection information. Nominal responses were required on a three-point scale (yes, no, and I do not know). For all nine items response I do not know was considered no. 

 The self-efficacy was assessed with eight items. Participants were asked about their confidence to engage in sun protection behaviors when out in the sun for 15 minutes or more. Responses to items on the self-efficacy were given on a scale ranged from cannot do at all (0) to certainly can do (10). 

### 2.4. Statistical Analysis

All data were analyzed using computerized SPSS version 21. Descriptive statistics were computed to characterize all the variables. Additionally, a Pearson correlation was performed to assess the associations between HBM constructs and sun protection behaviors. Alpha level of .05 was set a priori.

## 3. Results and Discussion

A total of 23 landscaping companies consisting of 140 employees were identified in North Mississippi. Only one company chose to have an investigator to administer the survey on site, which yielded six completed questionnaires. The remaining 134 questionnaires were mailed to 22 companies. Finally, data from 109 fully completed questionnaires were used for the analyses.

### 3.1. Description of Study Participants

 In this study, landscapers received substantial (*M* = 5.36 hours) sun exposure each day during the highest sun intensity hours (between 10:00 a.m. and 4:00 p.m.), which is higher than the amount of daily sun exposure reported by outdoor workers in the prior studies [2, 12, 17, 18]. It is noteworthy that none of the aforementioned previous studies have noted outdoor workers' level of sun exposure during the peak sun hours. Furthermore, consistent with the Salas et al.'s (2005) study on California farm workers, landscapers reported an average of 11.04 years of working outdoors, indicating long-term occupational exposure to sunlight [14]. The present study's results of regular high levels and chronic solar exposures are alarming when considering that 77.1% of the landscapers were White, approximately 50% had a skin type with a high propensity to burn rather than tan, 53.2% had light colored eye, and 22% revealed having light colored hair. 

### 3.2. Personal History of Skin Cancer

The skin cancer history rate (5.5%) reported by landscapers of this study is reasonably similar to the rate noted in the previous study (7%) [10]. However, this difference between the rates may be explained by the fact that mean age in the Marlenga's (1995) study was 50.88 years, with an average of 42.95 years of occupational sun exposure [10], while in the present study, mean age was 37.06 years, with an average of 11.04 years of occupational sun exposure. A significant issue to be considered in future research is occupational exposure of landscapers to insecticides/arsenic. This may be one of the reasons for the skin cancer history rates in this population.

### 3.3. Family History of Skin Cancer

 An interesting finding to emerge from the data comparisons was that family history of skin cancer in this study was higher than the rates revealed by Wisconsin dairy farmers (25.7% versus 15%) [10], Southern California postal workers (25.7% versus 17.7%) [17], and outdoor workers in San Diego County, California (25.7% versus 17.5%) [19]. One possible reason for different rates is differences in the questions used in the studies to determine family history of the skin cancer. Compared with the abovementioned studies, this study provided a more thorough definition of a family history of skin cancer (i.e., skin cancer in first degree relative: mother, father, brother, sister, or child); thereby, this finding supports the notion that it is crucial to take adequate definition of the variable into consideration while designing a question [20].

### 3.4. Sunburns

 With regard to sunburns, results revealed that more than half (58%) of the landscapers experienced at least one or more episodes of sunburns within the year preceding the survey; this shows that sun exposure level was high enough to induce sunburn. This study did not identify the sunburned body sites. Nevertheless, another plausible explanation for high prevalence of sunburn is that the landscapers did not protect themselves as much as they could to reduce sun exposure.

### 3.5. Sun Protection Behaviors

 Landscapers indicated more routine (i.e., frequently/always) use of sunglasses (78%), in comparison with wearing sunscreen and sun protective clothing. A similar pattern was reported by Sydney construction site workers, Australia [21]. The finding of the present study regarding sunglasses use is particularly surprising because it considerably exceeded the use of other sun protection strategies, and it was higher than sunglasses use noted in the previous studies [14, 21, 22]. This reflects that the relatively higher use of sunglasses among landscapers may not be deliberate sun protection practice and can be attributed to either (a) wearing sunglasses as protection against occupational hazards or (b) social norms. Wearing protection for occupational hazards may also be a reason why outdoor workers would wear long pants and/or long sleeves. Further studies are needed to extend our understanding of the salient reasons that underlie sun protection behaviors among landscapers.

The current sun protection behavior data were also compared with Marlenga's (1995) study conducted on Wisconsin dairy farmers [10]. The landscapers' frequently or always use of long pants (57.9%) was lower than that found in Wisconsin dairy farmers (90%), whereas use of sunscreen (28.4%) and long sleeved shirt (13.8%) was higher than those reported by Wisconsin dairy farmers (8% and 7%, resp.) [10]. Furthermore, for the use of gloves (15.6%) and wide-brimmed hat (14.7%), findings were in accordance with Marlenga (1995) (14% and 13%, resp.) [10]. Overall, however, landscapers' frequency to engage in sun protection behavior was not as high as would be optimal when working outdoors in summer between 10:00 a.m. and 4:00 p.m. Hence, the present study confirms the previous recommendations that there is a strong need to increase skin cancer prevention practices among outdoor workers [2, 10, 13, 14, 19].  Table 1 illustrates frequencies and percentages of sun protection behaviors. 

### 3.6. Knowledge and Health Beliefs

Overall, landscapers indicated mean score of 67.1% correct on the knowledge questions regarding skin cancer (see Table 3). This finding was slightly lower than that of a previous study (70%) [10]. Furthermore, with regard to health beliefs, the current findings are somewhat in line with results of an earlier study [10]. According to the HBM, an individual's perceived risk (i.e., perceived susceptibility and perceived severity), perceived benefits, and perceived barriers are partly dependent on his or her knowledge level [8]. Knowledge that sun exposure causes most skin cancers demonstrated the greatest number of correct responses (79.8%), which means that landscapers knew that the sun is a primary risk factor for skin cancer. However, 44.9% of the landscapers perceive that they were likely to get skin cancer sometime during their lifetime. On the contrary, 69.7% of landscapers perceived that they are more susceptible than the average person to get skin cancer. It is probable that the majority (69.7%) of landscapers perceived their likelihood to develop skin cancer higher than others, as they know that their sun exposure is higher compared to others. However, less than half (44.9%) perceived themselves likely to develop skin cancer indicating that they might not know about genetic risk factors which are responsible for skin cancer. Another likely reason behind the minority (44.9%) of landscapers perceiving that they are susceptible to skin cancer could be explained by low rate of personal skin cancer history. It is evident that individuals generally do not believe that they are at risk for disease, until they experience it themselves [8]. Future research should investigate landscapers' knowledge of other potential risk factors (i.e., genetic and personal) of skin cancer. 

 Likewise, results of the responses to perceived severity questions were mixed. Most landscapers (87.2%) agreed with the statement that “skin cancer is a serious disease,” whereas, only 13.8% agreed with the statement that “if they get skin cancer, they will not be able to continue work as a landscaper.” This may be explained by inconsistency in answers to knowledge questions. On the one hand, 73.4% of landscapers correctly identified that “skin cancer can cause death.” On the other hand, 43.1% thought “melanoma was the least serious form of skin cancer.” More broadly, additional studies are required to ascertain these speculations. In the workplace, an educational program that covers not only the seriousness of skin cancer but also the effects that skin cancer would have on one's ability to work is necessary. Our data reveal that people have not been well educated on the overall aspects of skin cancer. In fact, knowing where outdoor workers get their health knowledge (television, magazine articles, etc.) is helpful in preparing an educational program.

 Furthermore, the correlation analysis revealed no significant relationship between perceived threat and sun protection behaviors among this sample of landscapers (*r* = .001, *P* = .993). In contrast, Hammond et al. (2008) found that perceived risk of skin cancer led to an increase in sun protection practice. Nevertheless, Hammond and colleagues assessed perceived risk with single item measure and major weakness of their study was that reliability and validity of the instrument was not tested [11]. Hence, finding of our study may better capture the relationship of perceived threat with sun protection behaviors.

 Nearly three-quarters (73.4%) of landscapers correctly reported that “most skin cancers can be prevented.” This may have led the majority of the landscapers to believe that the use of wide-brimmed hat (60.5%), long sleeved shirt (69.7%), long pants (69.7%), and sunscreen (69.7%) are beneficial. In addition to this, 76.1% reported that “if they protect themselves from the sun daily, they will be less likely to get skin cancer.” Nevertheless, it was also found that 52.3% of the participants agreed or strongly agreed that tanned individuals look more attractive than individuals with no tan. Perhaps this landscapers' perceived attractiveness of tanned look is one of the barriers to the use of sun protection methods. It was documented that for many, the belief about physical attractiveness for tan skin contributes to unprotected exposure to sun for extended time periods [23, 24]. Given that landscapers receive significant amount of sun exposure at work, further researches should attempt to investigate both indoor and outdoor tanning behaviors as well as psychosocial factors associated with these behaviors of landscapers.

The fact that is the most commonly expressed barrier to wearing sun protective clothing was “it is too hot to wear” consistent with the previous studies [10, 25]. Regarding barriers to sunscreen and wide-brimmed hat use, present findings corroborate Marlenga (1995) who noted that the most frequently named barrier to continuous use of sunscreen and wide-brimmed hat was “I forget to wear it” [10]. On the other hand, results revealed that the “too much cost” of sun protection measures was the least commonly mentioned barrier, which is in accordance with previous studies and suggests that the affordability of sun protection is not a great concern among landscapers [10, 25, 26]. Barriers to sun protection behaviors are reported in Table 2.

 Moreover, results from the current study showed significant correlation between perceived benefits minus perceived barriers and sun protection behaviors (*r* = .285, *P* = .003). In order to enhance sun protection, landscapers should be educated about benefits of sun protection measures. At the same time, intervention programs should attempt to reduce landscapers' personal barriers to sun protection. In particular, this study did not attempt to collect information about landscapers' clothing fabric and fitting; therefore, it is difficult to make any inferences regarding why majority of the landscapers reported that sun protective clothing is “too hot to wear.” However, education of fabric characteristic and fitting should be considered when designing interventions focusing on barriers that may prevent landscapers engaging in sun protection [27]. Since another most commonly cited barrier to sun protection behaviors was “I forget to wear,” development of intervention strategies should consider verbal or visual reminder strategies which may trigger sun protection behaviors among landscapers. Family members should be encouraged to remind landscapers to use sun protection behaviors. Another strategy could be placing labels in landscapers' vehicles and on working instruments as reminder for sun protection. Cues to action such as these can be helpful reminders in the workplace. Another suggestion is to encourage companies to adopt a “sunscreen application break” policy, so the application of sunscreen is promoted. This would possibly prompt the outdoor worker to also make sure they have on a wide-brimmed hat and sunglasses. One of the reasons listed by the majority of landscapers for not using sunscreen was that it is greasy and smells bad. Health education professionals should ensure that landscapers know about the availability of sunscreen brands which are not oily and come in different fragrances. In fact, providing samples in the workplace of nonoily sunscreen brands would be beneficial to allow workers to try new or different products.

### 3.7. Cues to Action

 In the present study, the most frequently identified source of sun protection information was friends or family (78%). Parrott and Lemieux (2003) reported that skin cancer prevention and detection information given by families contributes to likelihood of farmers' sunscreen use [28]. Therefore, future sun protection education intervention should target not only landscapers but also their family members. However, the role of landscapers' families or friends in their sun protection activities should be explored in future research and this may also provide a clearer picture of social support in the landscapers' sun protection behaviors. 

 The majority of the landscapers in the present study also listed television (72.5%), magazine articles or advertisements (60.6%), and health information pamphlets (52.3%) as their common sources of information to protect from sun. Studies demonstrated that utilization of media channels to design an intervention can be an effective approach to increase the sun protection behaviors and reduce the risk of skin cancer among outdoor workers [29, 30]. An encouraging finding of our study was that 61.5% of the landscapers are receiving sun protection information from their doctor or other health care workers, indicating that landscapers are exposed to health care professionals. Based on the present study findings, it is suggested that partnerships between health care providers and media would be beneficial to disseminate sun protection information faster and maximize the access to large population of landscapers. Moreover, nurses and general physicians should be encouraged to educate their patients who work outdoors about sun protection and regular full body screening for skin cancer. The sun protection related counseling by health care providers has been shown to be effective in increasing outdoor workers' skin cancer prevention practices and knowledge [31]. 

### 3.8. Self-Efficacy

 Of all participants, 42.2% chose “cannot do at all (0)” in regard to the statement “limit sun exposure between 10 a.m. and 4 p.m.” About one-quarter reported “moderately certain can do (5)” regarding their confidence to wear wide-brimmed hat (25.7%) and work gloves (22%). Around one-fifth (19.3%) indicated confidence (10) to wear a long sleeved shirt. A total of 23% were confident to wear a sunscreen with a sun protection factor (SPF). The majority (42.2%) were confident (10) in their ability to wear long pants. Over half (54.1%) were confident (10) about wearing sunglasses.

 We found a significant relationship between self-efficacy and sun protection behaviors (*r* = .538, *P* = .001), suggesting that the higher the self-efficacy to engage in sun protections, the higher the likelihood of sun protection practices. Intervention programs that focus on increasing levels of sun protection should include strategies to increase self-efficacy to participate in sun protection behaviors. Future studies should apply Bandura's (1977) self-efficacy model (i.e., performance attainment, vicarious experiences, verbal persuasion, and physiological arousal) to identify the strategies [9].

## 4. Limitations

 There were several limitations to this study that should be acknowledged. First, although this study had a respectable response rate (83.6%) and data were collected from multiple geographical locations of Northern Mississippi, the sample was relatively small in size. Consequently, findings may not be generalizable to all landscapers of North Mississippi or other parts of the US. Second, most of the landscapers who participated were White (77.1%) and males (94.5%), limiting generalizability of the results to females and other racial/ethnic populations. Third, because this study used a convenience sampling, the possibility of self-selection bias cannot be ruled out. 

 The self-reported data of this study is subject to recall and social desirability biases. Another limitation of this study lies in the fact that internal consistency of the self-efficacy to engage in sun protection behavior was *α* = .55, questioning the internal consistency reliability across self-efficacy items.

We did not provide information or definitions about different forms of skin cancer. It is possible that landscapers misclassified personal or familial history of skin cancer with conditions such as seborrheic keratosis (SKs) or actinic keratosis (AKs) or moles removed that are not skin cancers.

 Furthermore, no attempt was made to control the influence of potential confounding variables. Therefore, caution must be applied when interpreting the results of relationships between HBM variables and practice of sun protection behavior. Also, the cross-sectional design of this study presents additional limitation that restricts causal relationships. 

## 5. Conclusion

The HBM proposes that individual's likelihood to engage in protective behavior is based on perceived threat. This HBM proposition is not supported in the present study. However, the results indicate that the difference of perceived benefits outweighing perceived barriers to sun protection is associated with sun protection behaviors. Furthermore, self-efficacy to engage in sun protection is associated with sun protection behaviors. The factors that account for absence of the relationship between perceived skin cancer threat and sun protection behaviors could be lack of skin cancer knowledge and low rate of personal skin cancer history. To better represent all landscapers in North Mississippi, a randomized study that incorporates a larger sample of landscapers is recommended. Certainly, a prospective design should be considered for future studies in order to provide more definitive evidence of directionality or causality between HBM variables and sun protection behaviors. In order to supplement information on barriers to sun protection practice, it is suggested that public health professionals should collaborate with local and state health and safety legislators to obtain information and to have an understanding of current policies and responsibilities of landscapers. Further research regarding the use of policy and provision of sun protection measures is warranted for this population.

## Figures and Tables

**Table 1:** Frequencies and percentages of sun protection behaviors.

Practice	Never *n* (%)	Rarely *n* (%)	Sometimes *n* (%)	Frequently *n* (%)	Always *n* (%)
Wear wide-brimmed hat	26 (23.9%)	31 (28.4%)	36 (33%)	11 (10.1%)	5 (4.6%)
Wear long sleeved shirt	40 (36.7%)	26 (23.9%)	28 (25.7%)	6 (5.5%)	9 (8.3%)
Wear long pants	9 (8.3%)	8 (7.3%)	29 (26.6%)	19 (17.4%)	44 (40.5%)
Wear work gloves	26 (23.9%)	29 (26.6%)	36 (33%)	13 (11.9%)	4 (3.7%)
Wear sunglasses	5 (4.6%)	6 (5.5%)	13 (11.9%)	27 (24.8%)	58 (53.2%)
Wear sunscreen	39 (35.8%)	17 (15.6%)	22 (20.2%)	25 (22.9%)	6 (5.5%)

**Table 2:** Frequencies and percentages of barriers to continuous practicing of sun protection behavior.

Barrier	Wide-brimmed hat *n* (%)	Long sleeved shirt *n* (%)	Long pants *n* (%)	Work gloves *n* (%)	Sunglasses *n* (%)	Sunscreen *n* (%)
Takes too much time	2 (1.8%)	1 (0.9%)	0 (0%)	4 (3.7%)	0 (0%)	23 (21.1%)
Inconvenient	46 (42.2%)	12 (11%)	6 (5.5%)	50 (45.9%)	12 (11%)	4 (3.7%)
Costs too much	1 (0.9%)	1 (0.9%)	0 (0%)	1 (0.9%)	0 (0%)	7 (6.4%)
Too hot to wear	28 (25.7%)	86 (78.9%)	60 (55%)	40 (36.7%)	3 (2.8%)	9 (8.3%)
Forget to wear	50 (45.9%)	11 (10.1%)	11 (10.1%)	52 (47.7%)	35 (32.1%)	70 (64.2%)

**Table 3:** Frequencies and percentages of correct responses to knowledge of skin cancer (descending order).

Statements	Correct responses *n* (%)
Sun exposure causes most skin cancers	87 (79.8%)
Most skin cancers can be prevented	80 (73.4%)
Skin cancer can cause death	80 (73.4%)
When skin cancer is detected early, the cure rate is very high	78 (71.6%)
The sun's rays are the strongest at midday	77 (70.6%)
A person with fair skin color needs the most protection from the sun	74 (67.9%)
Sunburn causes lasting damage to the skin	74 (67.9%)
Experts suggest using sunscreen with a sun protection factor (SPF) of 15 or higher	73 (67%)
Skin cancer is the most common form of cancer	55 (50.5%)
Melanoma is the least serious form of skin cancer	47 (43.1%)
